# Supplementary material for: Impact of a team-based versus individual clinician-focused training approach on primary healthcare professionals’ intention to have serious illness conversations with patients: A theory-informed process evaluation embedded within a cluster randomized trial
Source: PLoS One. 2025 Mar 26;20(3):e0298994. doi: 10.1371/journal.pone.0298994 (PMC11940443; doi:10.1371/journal.pone.0298994)
Supplement: S1 Fig — (DOCX) [file pone.0298994.s001.docx]

**S1 Fig: The COM-B model and its correlation with the TDF domains (50)**


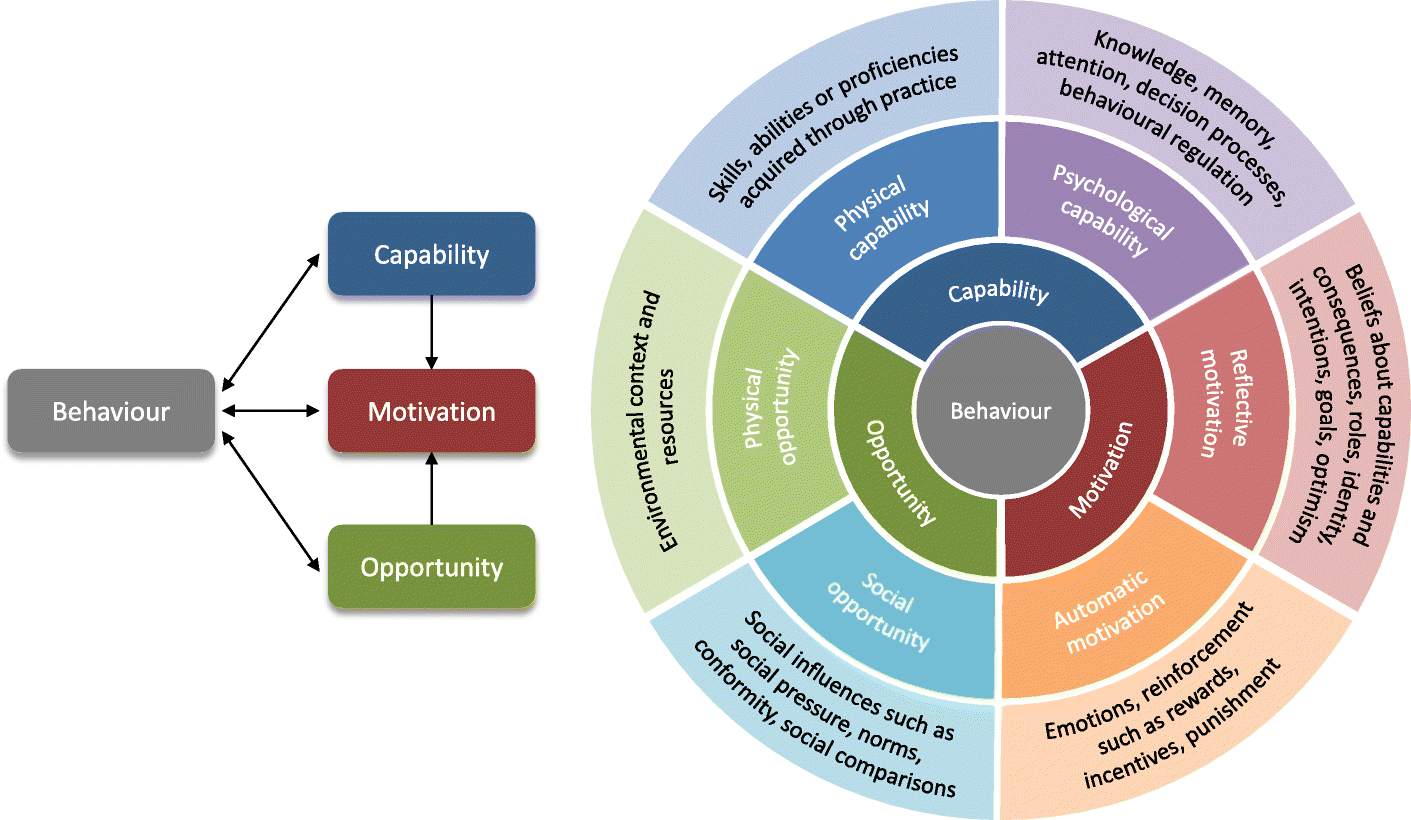


Image obtained at: McDonagh, L.K., Saunders, J.M., Cassell, J. *et al.* Application of the COM-B model to barriers and facilitators to chlamydia testing in general practice for young people and primary care practitioners: a systematic review. *Implementation Sci* **13**, 130 (2018). <https://doi.org/10.1186/s13012-018-0821-y> under the terms of the Creative Commons Attribution 4.0 International License (http://creativecommons.org/licenses/by/4.0/), which permits unrestricted use, distribution, and reproduction in any medium, provided you give appropriate credit to the original author(s) and the source.
