# Supplementary material for: Impact of a team-based versus individual clinician-focused training approach on primary healthcare professionals’ intention to have serious illness conversations with patients: A theory-informed process evaluation embedded within a cluster randomized trial
Source: PLoS One. 2025 Mar 26;20(3):e0298994. doi: 10.1371/journal.pone.0298994 (PMC11940443; doi:10.1371/journal.pone.0298994)
Supplement: S1 Table — (DOCX) [file pone.0298994.s002.docx]

**S1 Table: ACP engagement scores before and after training:**

|  | Mean (SE) | | Estimate differences^a^ | 95% Cl | P-Value |
| --- | --- | --- | --- | --- | --- |
|  | Team-based arm | Individual clinician-focused arm |  |  |  |
| How likely were you to engage patients in ACP before training? | 5.03 (0.26) | 6.11 (0.31) | -1.09 | -1.91; -0.26 | **0.01** |
| How likely are you to engage patients in ACP after training? | 7.95 (0.18) | 8.71 (0.21) | -0.75 | -1.32; -0.19 | **0.01** |

Analyzed using a Linear mixed model

P-value < 0.05 – statistically significant

95% CI, confidence interval at 95%**;** SE, standard error of the mean

^a^ Least squares mean
