# Supplementary material for: Impact of a team-based versus individual clinician-focused training approach on primary healthcare professionals’ intention to have serious illness conversations with patients: A theory-informed process evaluation embedded within a cluster randomized trial
Source: PLoS One. 2025 Mar 26;20(3):e0298994. doi: 10.1371/journal.pone.0298994 (PMC11940443; doi:10.1371/journal.pone.0298994)
Supplement: S2 Table — (DOCX) [file pone.0298994.s003.docx]

**S2 Table.** **Recommendations for improving the training based on barriers and facilitators, using the COM-B model, the Theoretical Domains Framework and the CPD-Reaction questionnaire.**

| **COM-B criteria sub-category** | **TDF Domains linked to COM-B** | **Barriers and facilitators** | **Psychosocial determinants of the CPD-Reaction questionnaire** | **Recommendations (COM-B intervention functions)** |
| --- | --- | --- | --- | --- |
| **Capability** | | | | |
| Psychological capability | Knowledge | PHCPs not knowing their patients and the difficulty in identifying the patients who would benefit. (barrier)  PHCPs knowing the patients and more able to identify those who needs serious illness conversations. (facilitator) |  | Enhance the team's understanding by offering additional education on identifying patients at increased risk who would benefit from a serious illness conversation (education).  Train PHCPs to understand the importance of early and timely serious illness conversations even with new patients (education). |
|  | Cognitive and interpersonal skills | More training, practice scenarios and standardized patients (Facilitator)  Lack of experience (barrier) |  | Provide more practice scenarios with standardized patients (education)  Assign a designated team champion for each role to exemplify the strategies that could be employed to have serious illness conversations and how to facilitate those conversations (modelling) |
|  | Behavioural regulation | Holding multiple conversations (facilitator) |  | Enable professionals to organize their schedule allowing for multiple conversations with patients (enablement) |
| **Opportunity** | | | | |
| Social opportunity | Social influences | All team members understand the relevance of serious illness conversations and help each other (facilitator)  Involving and coordinating multiple personnel in the process (barrier)  Lack of interprofessional communication about SICP (barrier)  Patient who are receptive and ready to talk (facilitator)  Communication issues with patients (barrier) | Social Influence | Include clinic group discussions to reevaluate the involvement of various team members and clarify their respective roles and difficulties during implementation (environmental restructuring)  Incorporate additional role-play sessions to ensure that all team members feel comfortable and confident in the roles they are assigned to perform in the real-world (Modelling) |
| Physical Opportunity | Environmental context and resources | Having the Serious Illness Conversations Guide (SICG) (facilitator)  Brief cheatsheet/buzzwords (instead of/in addition to the SICG) (facilitator)  Scheduled time designated for serious illness conversations (facilitator)  Having a clear workflow (facilitator)  Workflow adjustments(barrier)  Fitting Serious illness conversations into clinical schedule (barrier)  Difficulty to adapt the intervention to clinical routine (barrier)  Guides and templates are embedded in EHRs (facilitator)  More time to have these discussions (facilitator)  Unspecified barriers related to time (barrier)  Documenting conversations (barrier) |  | Make the guide available and accessible in all practices (environmental restructuring)  Adapt the guide to make a simpler version to facilitate discussions (environmental restructuring)  Adapt the EHR to include a section for serious illness conversations (environmental restructuring)  Enable professionals to create a workflow to have the discussion, share tasks that is adapted to their reality (enablement)  Adapt clinical schedules to allow multiple professionals to have dedicated time to have serious illness conversations (environmental restructuring)  Train professionals on how to document the conversations (training) |
| **Motivation** | | | | |
| Automatic motivation | Reinforcements | Being reminded to have serious illness conversations (facilitator)  Ongoing support (facilitator)  Billing (barrier and facilitator) |  | Restructure the environment to incorporate automatic reminders for initiating serious illness conversations (environmental restructuring)  Conduct follow-up training sessions with teams is crucial to address any lingering questions, provide clarification, and gain a deeper understanding of their experiences during discussions (education)  Revise the co-payment system to facilitate increased frequency of consultations for patients facing serious illnesses. (environmental restructuring) |
|  | Emotions | Discomfort with serious illness conversations and its emotional burden |  | Enhance the training by incorporating additional examples that demonstrate how structured discussions can positively impact both patients and professionals (persuasion) |
| Reflective Motivations | Beliefs about consequences | Patient’s response (barrier) | Beliefs about consequences | Include more patient and family testimonials in the training to emphasize that the general response to serious illness conversations is positive (persuasion) |
|  | Beliefs about capabilities | Develop more confidence and experience to have serious illness conversations. (facilitator) | Beliefs about capabilities | Integrate into the training of PHCPs to act as role models, explaining and giving cues on how to have serious illness conversations. (modelling) |
|  | Social/professional role and identity | Scope of practice (barrier) |  | Include more details in the training on which supportive roles can be performed by other team members who do not have as much direct contact with the patient (education) |
|  | Goals | Reaching out to patients (barrier) |  | Improve role-play with standardized patients to help professionals practice reaching out to patients (education) |
