# Supplementary material for: Impact of a team-based versus individual clinician-focused training approach on primary healthcare professionals’ intention to have serious illness conversations with patients: A theory-informed process evaluation embedded within a cluster randomized trial
Source: PLoS One. 2025 Mar 26;20(3):e0298994. doi: 10.1371/journal.pone.0298994 (PMC11940443; doi:10.1371/journal.pone.0298994)
Supplement: S1 Checklist — (DOCX) [file pone.0298994.s004.docx]

# S1 Checklist: CONSORT 2010 checklist of information to include when reporting a cluster randomised trial

| **Section/Topic** | **Item No** | **Standard Checklist item** | **Extension for cluster designs** | **Page No *** |
| --- | --- | --- | --- | --- |
| **Title and abstract** | | | | |
|  | 1a | Identification as a randomised trial in the title | Identification as a cluster randomised trial in the title | Page 1 |
|  | 1b | Structured summary of trial design, methods, results, and conclusions (for specific guidance see CONSORT for abstracts)1,2 | See table 2 | Page 3 |
| **Introduction** | | | | |
| **Background and** | 2a | Scientific background and explanation of rationale | Rationale for using a cluster design | Pages 4-5 |
| **objectives** |  |  |  |  |
|  | 2b | Specific objectives or hypotheses | Whether objectives pertain to the the cluster level, the individual participant level or both | Page 5 |
| **Methods** | | | | |
| **Trial design** | 3a | Description of trial design (such as parallel, factorial) including allocation ratio | Definition of cluster and description of how the design features apply to the clusters | Page 5 |
|  | 3b | Important changes to methods after trial commencement (such as eligibility criteria), with reasons |  | **N/A** |
| **Participants** | 4a | Eligibility criteria for participants | Eligibility criteria for clusters | Page 6 |
|  | 4b | Settings and locations where the data were collected |  | Page 9 |
| **Interventions** | 5 | The interventions for each group with sufficient details to allow replication, including how and when they were actually administered | Whether interventions pertain to the cluster level, the individual participant level or both | Pages 6 and 7 |
| **Outcomes** | 6a | Completely defined pre- specified primary and secondary outcome  measures, including how and | Whether outcome measures pertain to the cluster level, the individual participant level or both | Pages 7 and 8 |

| when they were assessed | | | | |
| --- | --- | --- | --- | --- |
|  | 6b | Any changes to trial outcomes after the trial commenced, with reasons |  | **N/A** |
| **Sample size** | 7a | How sample size was determined | Method of calculation, number of clusters(s) (and whether equal or unequal cluster sizes are assumed), cluster size, a coefficient of intracluster correlation (ICC or *k*), and an indication of its uncertainty | Page 8 |
|  | 7b | When applicable, explanation of any interim analyses and stopping guidelines |  | **N/A** |
| **Randomisation:** | | | | |
| **Sequence generation** | 8a | Method used to generate the random allocation sequence |  | **Page 6** |
|  | 8b | Type of randomisation; details of any restriction (such as blocking and block size) | Details of stratification or matching if used | Page 6 |
| **Allocation concealment mechanism** | 9 | Mechanism used to implement the random allocation sequence (such as sequentially numbered containers), describing any steps taken to conceal the sequence until interventions were assigned | Specification that allocation was based on clusters rather than individuals and whether allocation concealment (if any) was at the cluster level, the individual participant level or both | Page 6 |
| **Implementation** | 10 | Who generated the random allocation sequence, who enrolled participants, and who assigned participants to interventions | Replace by 10a, 10b and 10c | Pages 6 and 7 |
|  | 10a |  | Who generated the random allocation sequence, who enrolled clusters, and who assigned clusters to interventions | Pages 6 and 7 |
|  | 10b |  | Mechanism by which individual participants were included in clusters for the purposes of the trial (such as complete enumeration, random sampling) | Pages 6 and 7 |

|  | 10c | From whom consent was sought Not detailed (representatives of the cluster, or (information in individual cluster members, or the published both), and whether consent was protocol)  sought before or after randomisation | |
| --- | --- | --- | --- |
|  | | | |
| **Blinding** | 11a | If done, who was blinded after assignment to interventions (for example, participants, care providers, those assessing outcomes) and how | Pages 6 and 7 |
|  | 11b | If relevant, description of the similarity of interventions | Pages 7 and 8 |
| **Statistical methods** | 12a | Statistical methods used to How clustering was taken into compare groups for primary account  and secondary outcomes | Pages 9-10 |
|  | 12b | Methods for additional analyses, such as subgroup analyses and adjusted analyses | Pages 9-10 |
| **Results** | | | |
| **Participant flow (a diagram is strongly recommended)** | 13a | For each group, the numbers For each group, the numbers of of participants who were clusters that were randomly randomly assigned, received assigned, received intended intended treatment, and treatment, and were analysed for were analysed for the the primary outcome  primary outcome | Pages 10 and  figure 2 |
|  | 13b | For each group, losses and For each group, losses and exclusions after exclusions for both clusters and randomisation, together with individual cluster members reasons | Figure 2 |
| **Recruitment** | 14a | Dates defining the periods of recruitment and follow-up | Page 7 |
|  | 14b | Why the trial ended or was stopped | N/A |
| **Baseline data** | 15 | A table showing baseline Baseline characteristics for the demographic and clinical individual and cluster levels as characteristics for each applicable for each group group | Pages 10-13 |

| **Numbers analysed** | 16 | For each group, number of participants (denominator) included in each analysis and whether the analysis was by original assigned groups | For each group, number of clusters included in each analysis | Pages 10 and  figure 2 |
| --- | --- | --- | --- | --- |
| **Outcomes and** | 17a | For each primary and secondary outcome, results for each group, and the estimated effect size and its precision (such as 95% confidence interval) | Results at the individual or cluster level as applicable and a coefficient of intracluster correlation (ICC or k) for each primary outcome | Pages 13-21 |
| **estimation** |  |  |  |  |
|  | 17b | For binary outcomes, presentation of both absolute and relative effect sizes is recommended |  | N/A |
| **Ancillary analyses** | 18 | Results of any other analyses performed, including subgroup analyses and adjusted analyses, distinguishing pre-specified from exploratory |  | N/A |
| **Harms** | 19 | All important harms or unintended effects in each group (for specific guidance see CONSORT for harms3) |  | N/A |
| **Discussion** | | | | |
| **Limitations** | 20 | Trial limitations, addressing sources of potential bias, imprecision, and, if relevant, multiplicity of analyses |  | Page 24 |
| **Generalisability** | 21 | Generalisability (external validity, applicability) of the trial findings | Generalisability to clusters and/or individual participants (as relevant) | Pages 24 and 25 |
| **Interpretation** | 22 | Interpretation consistent with results, balancing benefits and harms, and considering other relevant evidence |  | Pages 21 to 24 |
| **Other information** | | | | |
| **Registration** | 23 | Registration number and name of trial registry |  | Page 6 |
| **Protocol** | 24 | Where the full trial protocol |  | Page 6 |

| can be accessed, if available | | | |
| --- | --- | --- | --- |
| **Funding** | 25 | Sources of funding and other support (such as supply of drugs), role of funders | Mentioned in the submission as required by the journal. |

** Note: page numbers optional depending on journal requirements*

**REFERENCES**

1 Hopewell S, Clarke M, Moher D, Wager E, Middleton P, Altman DG, et al. CONSORT for reporting randomised trials in journal and conference abstracts. *Lancet* 2008, 371:281-283

2 Hopewell S, Clarke M, Moher D, Wager E, Middleton P, Altman DG at al (2008) CONSORT for reporting randomized controlled trials in journal and conference abstracts: explanation and elaboration. *PLoS Med* 5(1): e20

^3^ Ioannidis JP, Evans SJ, Gotzsche PC, O'Neill RT, Altman DG, Schulz K, Moher D. Better reporting of harms in randomized trials: an extension of the CONSORT statement. *Ann Intern Med* 2004; 141(10):781-788.
