# Supplementary material for: Impact of a team-based versus individual clinician-focused training approach on primary healthcare professionals’ intention to have serious illness conversations with patients: A theory-informed process evaluation embedded within a cluster randomized trial
Source: PLoS One. 2025 Mar 26;20(3):e0298994. doi: 10.1371/journal.pone.0298994 (PMC11940443; doi:10.1371/journal.pone.0298994)
Supplement: S2 Checklist — (DOCX) [file pone.0298994.s005.docx]

S2 Checklist: Good Reporting of A Mixed Methods Study (GRAMMS) checklist

| **Guideline** | **Section: page** |
| --- | --- |
| Describe the justification for using a mixed methods approach to the research question | Methods- under Study design and settings page 5 |
| Describe the design in terms of the purpose, priority and sequence of methods | Methods  - outcomes pages 7-8 and 9-10 |
| Describe each method in terms of sampling, data collection and analysis | Data Collection, Sample size and analysis pages 9-10 |
| Describe where integration has occurred, how it has occurred and who has participated in it | Triangulating qualitative and quantitative data: page 10 |
| Describe any limitation of one method associated with the present of the other method | Discussion pages 24-25 |
| Describe any insights gained from mixing or integrating methods | Discussion: pages 21-24 |

O'Cathain A, Murphy E, Nicholl J. The quality of mixed methods studies in health services research. J Health Serv Res Policy. 2008;13: 92-98.
