# Supplementary material for: Impact of a team-based versus individual clinician-focused training approach on primary healthcare professionals’ intention to have serious illness conversations with patients: A theory-informed process evaluation embedded within a cluster randomized trial
Source: PLoS One. 2025 Mar 26;20(3):e0298994. doi: 10.1371/journal.pone.0298994 (PMC11940443; doi:10.1371/journal.pone.0298994)
Supplement: Attachment — Submitted filename: Response to Reviewers.docx [file pone.0298994.s007.docx]

| Dear reviewers,  Thank you for your valuable feedback on our paper. Your comments are greatly appreciated, and we have carefully considered each point raised. Below, you will find our detailed responses to address your suggestions: | | |
| --- | --- | --- |
| **Reviewer 1** | | |
| Comment | Response | Page |
| Although the the manuscript has the intention to evaluate the cRTs but the real outcome as per title is the intention serious illness conversations with patients (SICP) was not addressed in the background of abstract.  There is need to address the gaps and magnitude of the intention SICP as the problem statement in the introduction and more issues to be highlighted on the outcome. | Dear reviewer,  Thank you for this comment, which has helped us clarify in the paper how measuring PHCP intention functioned as a process evaluation. We have rewritten the background of the Abstract as follows:  “Cluster randomized trials (cRTs) on the effectiveness of training programs face complex challenges when conducted in real-world settings. Process evaluations embedded within cRTs can help explain their results by exploring possible causal mechanisms impacting training effectiveness”  We also added this paragraph in the Introduction to better explain this:  Process evaluations embedded within cRTs aim to provide insights into how an intervention was delivered, how participants received it, and whether it was implemented as intended (French, Pinnock et al. 2020). In the parent Meta-LARC ACP cRT, the impact of a team-based training approach compared to a clinician-based training approach was measured using patient centered outcomes (Totten, Fagnan et al. 2019). In this process evaluation, measuring the impact of the intervention on PHCPs themselves instead of patients and, specifically, on PHCPs’ intention to have serious illness conversations with patients was necessary to provide insights into how the team-based training approach was delivered, received and implemented in the parent cRT.  A process evaluation focusing on PHCPs behavioral intentions, and its psychosocial determinants would thus provide information about key factors affecting effective implementation of the intervention and could inform recommendations for its improvement. Key factors would include modifiable psychosocial factors that influence PHCPs’ intention to have serious illness conversations, and barriers and facilitators that they perceive to implementing the conversations. Thus, to influence behavior through behavioral intention, one would need to rely on modifying psychosocial variables using known behavior change techniques (Michie, Atkins et al. 2014)  We also added the following paragraph to highlight the knowledge gap that motivated our study and also detail the magnitude of intention  No studies have specifically addressed the factors that drive PHCPs’ intentions to have serious illness conversations with patients. Understanding these components is crucial for designing training interventions that effectively promote the adoption of this important behavior in clinical practice.  According to several socio-cognitive theories, behavioral intention is the central factor influencing the adoption of a given behavior and is therefore an acceptable surrogate for behavior change (Godin, Bélanger-Gravel et al. 2008, Hrisos, Eccles et al. 2009, Godin 2013, Légaré, Borduas et al. 2014, Bakwa Kanyinga, Gogovor et al. 2023). It provides a more immediate and cost-effective measure of actual clinical behavior change and is widely used to evaluate the efficacy of professional development training interventions (Goulet, Hudon et al. 2013). Measuring intention would thus allow for a targeted assessment of the intervention's influence on PHCPs, the primary implementers of the intervention in the parent cRT. It could also provide information on intervention strengths and weaknesses to inform the interpretation and adaptation of future SICP interventions (Grimshaw, Presseau et al. 2014). Ultimately, it could contribute to a cumulative implementation science knowledge base. | Pages 3 and 6 |
| Methodology was well elaborated, and the results were well discussed. | We appreciate your feedback. |  |
| **Reviewer 2** | | |
| Comment | Response: | Page |
| There is a need to shorten the article. manuscript. It appears rather difficult to read through. | Dear reviewer,  Thank you for your valuable feedback. We have shortened the manuscript considerably (approximately 800 words) and edited it for improved readability. We hope these changes make it easier to follow and understand. | - |
| Use academic language throughout the document. Refrain from usage of ‘we’ (as in abstract), repeated several times. | Thank you for your comment. We appreciate your suggestions for improving the academic tone of our manuscript. We've carefully reviewed your comments and revised the document to eliminate all first-person pronouns and avoid any informal language. | - |
| Figure S1 needs to be improved on or removed. | We appreciate your feedback regarding Figure S1. After careful consideration, we have decided to remove the figure. We believe that the information presented in the figure is sufficiently covered in the preceding paragraph, which cites references 17 and 18 to elaborate on how behaviors are embedded in a complex system. Additionally, we have provided a detailed explanation of how the variables were collected, ensuring that the necessary context is conveyed without the need for the supplemental figure. | Page 10 |
| The Meta-Larc Appendix has the term advanced care planning, whereas the title here is serious illness. | Dear reviewer,  Thank you for this comment. We agree that use of the two terms can be confusing. The title "Meta-LARC ACP" is the name consistently used to refer to the parent trial. This term was adopted in various scientific communications and is the trial's official name in current usage:  <https://doi.org/10.1016/j.jpainsymman.2022.04.019>  <https://doi.org/10.1016/j.jpainsymman.2023.02.297>  Doi: 10.35680/2372-0247.1808.  There is however an ongoing debate in palliative care concerning the terminology—whether "serious illness conversations" (SIC) or "advance care planning" (ACP) is more appropriate. (doi:10.1001/jama.2021.23695 or [10.1111/jgs.16801](https://doi.org/10.1111/jgs.16801))  The original trial that developed the Serious Illness Care Program framed serious illness conversations as a tool for ACP ([10.1136/bmjopen-2015-009032](https://doi.org/10.1136/bmjopen-2015-009032)).  However, to provide a more comprehensive understanding of behaviors related to end of life care our research incorporated both ACP and SIC as complementary concepts and behaviors. This approach avoids potential controversies and allows for a deeper analysis of the theme.  As Lou, Atkins, and West suggest (https://doi.org/10.1186/s13012-017-0605-9), behaviors are part of a broader system and cannot be isolated. By progressively broadening the scope of behaviors assessed, - in this case using both SIC and ACP - we succeeded in enhancing both the comprehensiveness and detail of our analysis.  We believe this explanation clarifies our rationale for combining ACP and SIC in our research.  However, to further clarify this difference for our readers, we have added the following sentence in the outcomes and measurements section (methods):  “ACP was included because it is a more established, broader concept in the field that encompasses end-of-life discussions, care objectives and their legal documentation (Sudore, Hickman et al. 2022).” |  |
| There is a need to improve on the clarity of the whole | Thank you for your valuable feedback. We’ve addressed your suggestion by having a professional scientific editor review the manuscript, enhancing its clarity, conciseness and academic tone. We believe these revisions have greatly improved readability and facilitated reader comprehension. | - |
